# Supplementary material for: Overexpression of the Gene Encoding Neurosecretory Protein GL Precursor Prevents Excessive Fat Accumulation in the Adipose Tissue of Mice Fed a Long-Term High-Fat Diet
Source: Molecules. 2021 Oct 3;26(19):6006. doi: 10.3390/molecules26196006 (PMC8512635; doi:10.3390/molecules26196006)
Supplement: Supplementary file 1 [file molecules-26-06006-s001.zip › molecules-1382822-supplementary.pdf]

molecules

Supplementary Materials

Overexpression of the Gene Encoding Neurosecretory Protein GL Precursor Prevents Excessive Fat Accumulation in the Adipose Tissue of Mice Fed a Long-Term High-Fat Diet

Keisuke Fukumura *,†, Yuki Narimatsu †, Shogo Moriwaki, Eiko Iwakoshi-Ukena, Megumi Furumitsu and Kazuyoshi Ukena *

Laboratory of Neurometabolism, Graduate School of Integrated Sciences for Life, Hiroshima University, Higashi-Hiroshima, Hiroshima 739-8521, Japan; d214243@hiroshima-u.ac.jp (Y.N.); m203300@hiroshima-u.ac.jp (S.M.); iwakoshi@hiroshima-u.ac.jp (E.I.-U.); mfurumi@hiroshima-u.ac.jp (M.F.)

* Correspondence: kfuku@hiroshima-u.ac.jp (K.F.); ukena@hiroshima-u.ac.jp (K.U.)

† These authors contributed equally to this work.

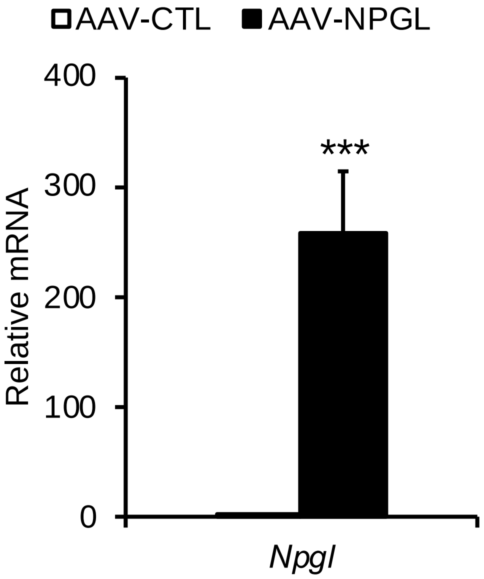

Figure S1. Intensity of *Npgl* overexpression at the experimental endpoint in the mediobasal hypothalamus of mice fed a high-fat diet (60% calories from fat). These mice were injected with an adeno-associated virus (AAV) vector, either a control (AAV-CTL) or a vector carrying the NPGL precursor gene (AAV-NPGL). The panel compares the levels of mRNA expression of *Npgl* between the AAV-CTL and AAV-NPGL groups. Statistical analysis was performed using Student's *t*-test. Each value represents the mean ± standard error of the mean (AAV-CTL: $n = 8$, AAV-NPGL: $n = 8$; ***$p < 0.005$).

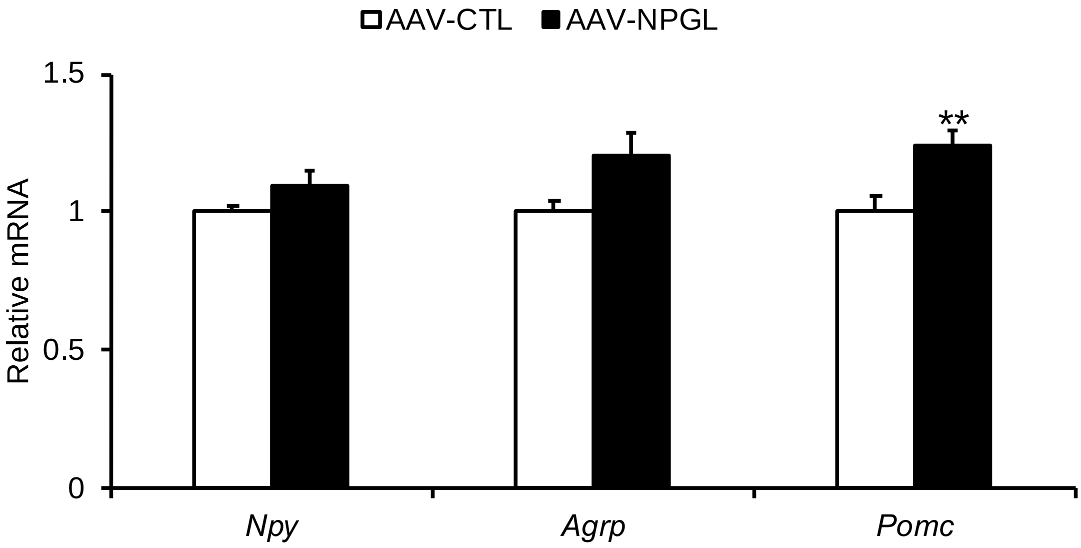

Figure S2. Effects of *Npgl* overexpression on mRNA expression of neuropeptides (*Npy*, neuropeptide Y; *Agrp*, agouti-related peptide) and a neuropeptide precursor (*Pomc*; proopiomelanocortin) in the mediobasal hypothalamus of mice fed a high-fat diet (60% calories from fat). These mice were injected with an adeno-associated virus (AAV) vector, either a control (AAV-CTL) or a vector carrying the NPGL precursor gene (AAV-NPGL). The panel compares the levels of mRNA expression of *Npy*, *Agrp*, and *Pomc* between the AAV-CTL and AAV-NPGL groups. Statistical analysis was performed using two-way ANOVA followed by Bonferroni's test. Each value represents the mean ± standard error of the mean (AAV-CTL: $n = 8$, AAV-NPGL: $n = 8$; $^{**}p < 0.01$).
